# Multispectral Photonic Structural Colors via Enhanced Interfacial Interference of Ultrathin Cellulose Nanofiber/MXene Films

**DOI:** 10.1002/advs.202500953

**Published:** 2025-04-07

**Authors:** Valeriia Poliukhova, Botyo Dimitrov, Justin Brackenridge, Laura Mae Killingsworth, Iryna Roslyk, James Fitzpatrick, Yury Gogotsi, Vladimir V. Tsukruk

**Affiliations:** ^1^ School of Materials Science and Engineering Georgia Institute of Technology Atlanta GA 30332 USA; ^2^ A. J. Drexel Nanomaterials Institute and Department of Materials Science and Engineering Drexel University Philadelphia PA 19104 USA

**Keywords:** cellulose nanofibers, layered composite, multilayer thin film interference, periodic structural colors, Ti_3_C_2_T_x_ MXene flakes

## Abstract

The study reports novel photonic properties of Ti_3_C_2_T*_x_* MXene flakes horizontally self‐assembled within cellulose nanofiber (CNF) matrix exhibiting unique bright multispectral colors combined with overall high transparency in the transmission regime. The intense reflection colors are reflected by individual flakes acting as effective micromirrors with shifts based on their subsurface positioning within the dielectric layers. Unique color appearances are controlled by an interplay of multiple bandgaps formed by constructive and destructive interferences at flake‐matrix interfaces. These colors manifest at the microscale under bright field optical microscopy, while the total physical film retains high transparency up to 85% and a typical greenish hue characteristic of the MXene content below 1% volume fraction. The diverse spectral appearance of 4 µm ultra‐thin films is ultimately controlled by the positioning of the horizontal flakes within the nanofiber matrix at diverse distances from the top surface. This work expands the understanding of thin films with assembled 2D materials within polymer matrix and their fundamental interactions creating new structural coloration functionalities with the potential for multispectral photonic applications such as camouflaging, photothermal treatment, and optical communication for flexible thin bio‐derived films.

## Graphical abstract

Fabrication of CNF‐MXene free‐standing films: Ti_3_C_2_T*_x_* MXene flakes integrated into a cellulose nanofiber (CNF) matrix through layered self‐assembly using vacuum‐assisted filtration. The layered structure of 4 µm thickness is displayed on SEM image. The schematic illustrates the structural colors of individual MXene flakes that change periodically within CNF layers involving complex light interaction.

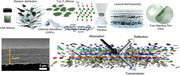

## 1 Introduction

In nature, vivid colors can be observed in the feathers of birds, butterfly wings, and scales of fish. These depend on the intricate microstructure of the surfaces and the complex interaction of light through diffraction, scattering, and interference, often resulting in iridescent or highly angle‐specific effects. Nature's manipulation of light within these structures is represented by typical optical processes that yield structural colors: thin‐film interference, multilayer interference, and photonic crystals.^[^ ^1^ ^]^ Multilayer interference is observed in thicker films with periodically stacked layers with high and low refractive index, where each layer is equal to λ/4, and the viewing angle is close to 90°.^[^ ^2^ ^]^ An ideal reflector is a ‘mother‐of‐pearl’ or a hierarchical nacre structure exhibiting iridescent colors in seashells, which are also used as armor to protect from predators.^[^ ^2^, ^3^ ^]^

MXenes, new inorganic 2D materials, are derived from layered transition metal carbides, typically by chemical etching, and exhibit a mix of properties that enhance their versatility and performance in diverse applications, from energy storage systems to electromagnetic interference shielding and wearable electronics.^[^ ^4^, ^5^ ^]^ The versatility of MXenes, in particular Ti_3_C_2_T*_x_*, allows for processing into various architectures, such as films, fibers, and aerogels, through methods like vacuum‐assisted filtration (VAF), spin‐coating, and spray‐coating to create high‐performance devices.^[^ ^6^, ^7^ ^]^ MXene/polymer combination opens the door to various nanocomposites with tailored properties that can improve electrical conductivity and mechanical strength when combined with the flexibility and processability of polymers.^[^ ^6^, ^8^ ^]^ Moreover, addressing challenges such as Ti_3_C_2_T*_x_* surface chemistry control and developing self‐assembly techniques is essential for fabricating flexible, free‐standing MXene films.^[^ ^5^, ^9^ ^]^ Surface functional groups like ─F, ─O, and ─OH allow MXene nanosheets to form diverse macrostructures through hydrogen bonding, preserving their inherent properties. Yet, MXene's tendency to restack and weak inter‐sheet connections may affect its performance.^[^ ^4^, ^10^ ^]^ Polymers containing hydrophilic polar groups can be introduced in MXene composites via hydrogen bonding interactions to produce high‐strength functional nanocomposites.^[^ ^10^, ^11^, ^12^ ^]^

Cellulose nanofibers (CNFs) are a natural polymer notable for unique structural and chemical properties exhibiting high mechanical strength, large surface area, and excellent dispersibility in water due to the introduction of carboxyl groups on the cellulose backbone during a typical synthetic procedure of TEMPO (2,2,6,6‐tetramethylpiperidine‐1‐oxyl) oxidation.^[^ ^10^, ^13^, ^14^ ^]^ These functional groups enhance the nanofibers' stability and compatibility with various matrices and impart anion‐exchange capabilities, making them ideal for water purification, biomedical applications, and as reinforcement materials in composites.^[^ ^15^, ^16^ ^]^ CNFs are biodegradable and derived from renewable resources, offering sustainable alternatives to synthetic polymers. Their ability to form strong hydrogen bonding results in transparent, flexible, and strong films, further underscores their superiority over conventional polymers in high‐performance applications.^[^ ^16^, ^17^, ^18^ ^]^

Hydrogen bonding, van der Waals forces, electrostatic interactions, and mechanical entanglement between random nanofibers and MXene nanosheets in the assembled films might result in versatile composites with enhanced strength.^[^ ^19^ ^]^ However, during the VAF of colloids with a high content of Ti_3_C_2_T_x_, the assembly of nanosheets relies on the face‐to‐face stacking of MXene flakes that align and bind preferentially with each other into a layered film when the solvent evaporates.^[^ ^20^ ^]^ Capillary forces further contribute to the restacking and random nature of flake assembly during the film formation.^[^ ^4^, ^21^ ^]^ For instance, additions of 60% and 20% CNFs in composite films resulted in a layered structure similar to pure MXene films with enhanced mechanical properties due to the reinforcement with nanofibers.^[^ ^21^ ^]^ While efforts have been made in CNF‐graphene oxide‐based composites with tailored functionalities,^[^ ^22^, ^23^, ^24^ ^]^ optical properties of CNF‐MXene materials have rarely been addressed.^[^ ^9^, ^18^ ^]^ Most conventional CNF‐MXene composites studied to date emphasize MXene's predominance for enhanced conductivity and mechanical strength.^[^ ^25^, ^26^, ^27^, ^28^, ^29^ ^]^

Focusing on Ti_3_C_2_T*_x_* MXene, a 2D material, and 1D cellulose nanofibers, as a random biodegradable natural polymer matrix, we delve into the fabrication of self‐assembled thin films with unexpected iridescence not shown by individual components.^[^ ^30^, ^31^ ^]^ By adapting MXene flake distribution within the cellulose matrix via assembling conditions, we observed a rich pattern of localized light‐matter interactions that induce the multispectral color appearance of individual flakes that vary periodically within their sub‐surface location. This layered self‐assembly mimics natural structural coloration mechanisms through constructive and destructive interference phenomena occurring in the cellulose nanofiber matrix with the metallic Ti_3_C_2_T*_x_* flakes. This research refocuses on exploring the impact of individual 2D flakes self‐assembled in various sub‐surface depths of cellulose nanofiber networks. Depending on the MXene concentration, resultant films can be produced without aggregation of the flakes, allowing one to distinguish a single flake within the cellulose network according to their distinct reflectance. Unexpectedly, these flakes exhibit high individual‐rich reflection in the visible range, while the film itself remains highly transparent in transmission mode. We demonstrated that Ti_3_C_2_T*_x_* flakes distributed in cellulose matrix within different dielectric distances in‐between the flakes and CNF surrounding, tailor rich reflective iridescent colors due to the interplay of optical interference of individual MXene “mirrors” of self‐assembled insulator‐metal‐insulator microcavities within these layered photonic films.

## 2 Results and Discussion

### 2.1 CNF‐MXene Free‐Standing Film Fabrication

**Figure 1:**
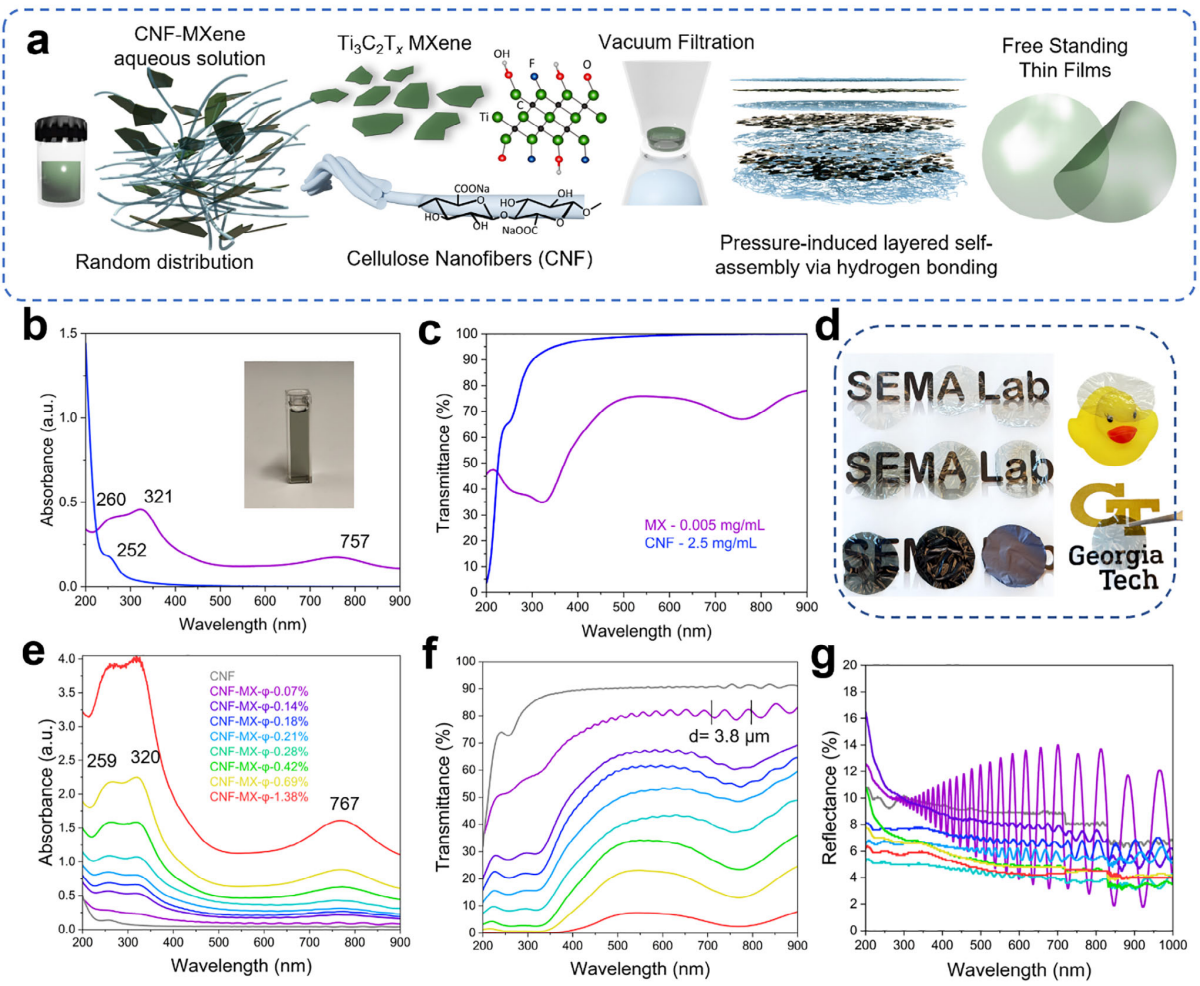
CNF‐MXene thin film fabrication and spectroscopic studies. a) Schematic illustration of the CNF‐MXene free‐standing films fabrication – Ti_3_C_2_T*_x_* MXene individual flakes organized within cellulose nanofiber (CNF) network in layered self‐assembly via vacuum‐assisted filtration (VAF). b,c) UV–vis–NIR absorbance, and transmittance spectra of Ti_3_C_2_T*_x_* and cellulose nanofibers colloidal solutions. d, Photographs of the obtained CNF, CNF‐MXene composite films and MXene film. e–g) Absorbance, transmittance, and reflectance spectra of the obtained CNF, CNF‐MXene films.

Hydroxyl groups of CNF and MXene's OH terminations facilitate hydrogen bonding by simply mixing the two components, forming a homogenous dispersion of the flakes within cellulose media (**Figure** **1**a).^[^ ^32^ ^]^ In this study, we considered different ratios between MXene and CNF in the preparation of the thin films in which MXene occupies < 2% volume fraction of the composite to avoid excessive light absorption (Tables S1 and S2, Supporting Information). Composite films are named respectively to the volume fraction *φ* of MXene, *CNF‐MX‐φ‐x*, where x is the respective *φ* value.

### 2.2 Spectroscopic Analysis

We first observe the absorbance and transmittance spectra of individual components, MXene and CNF, separately in colloidal dispersions (Figure 1b,c). As expected, Ti_3_C_2_T*_x_* shows typical absorbance peaks in the UV range at 260 nm, 321 nm, and NIR at 757 nm, while CNF has a strong absorbance at 200 nm with a shoulder at 252 nm.^[^ ^11^ ^]^ The absorbance and transmittance spectra of the CNF‐MXene films are plotted for different compositions (Figure 1e,f). The MXene content directly influences the light absorption properties of the composites, as summarized in Tables S1 and S3 (Supporting Information).^[^ ^33^ ^]^

Notably, when dispersed in water (Figure S1 and Table S3, Supporting Information), MXene's highest absorbance can be recorded up to 0.03 mg/mL, with the higher concentration mixtures of 0.05 mg mL^−1^ becoming too opaque to obtain the absorbance spectra. CNF‐MX films and dispersions absorb light in the UV region from 200 to 500 nm with a broad NIR absorption band around 700–900 nm (Figure 1e,f; Figure S1, Supporting Information), which reflects the greenish hue of the films (Figure 1d).

The optical transmittance is shown over the same wavelength range for the films and water‐based MXene dispersions (Figure 1f; Figure S1b, Supporting Information). CNF film maintains a high transmittance of ≈90% across the visible spectrum, with the transmittance decreasing with MX content increasing (Figure 1f; Table S1, Supporting Information). The transmittance in composite CNF‐MXene films is generally higher than in CNF‐MXene dispersions recorded before film fabrication (Tables S1 and S3, Supporting Information).

Another intriguing, unexpected observation for films with the lowest MXene content, from 0.07% up to 0.42% *φ*, is the presence of strong, periodic interference at both air‐film interfaces (Figure 1f).^[^ ^2^ ^]^ These interference effects are caused by light reflection at air‐film interfaces, indicating smooth surfaces, high film uniformity, and high effective refractive properties of nearly transparent films. Indeed, the thickness of the *CNF‐MX‐φ‐0.07%* film calculated from periodic peaks and composite refractive index of 1.46 (as obtained from ellipsometry) closely matches the 4.0 µm film thickness observed in SEM images (Figures S2, S3, Supporting Information). As a result of this phenomenon, strong iridescent colors are also observed when the film is viewed under white light, as different wavelengths are amplified or diminished (Figure 1d). Reflection measurements further confirmed extremely strong optical interference reaching 10–14% and especially amplified for the lowest MXene content (Figure 1g).

### 2.3 Morphology, Surface, and Composition of CNF‐MX Films

**Figure 2:**
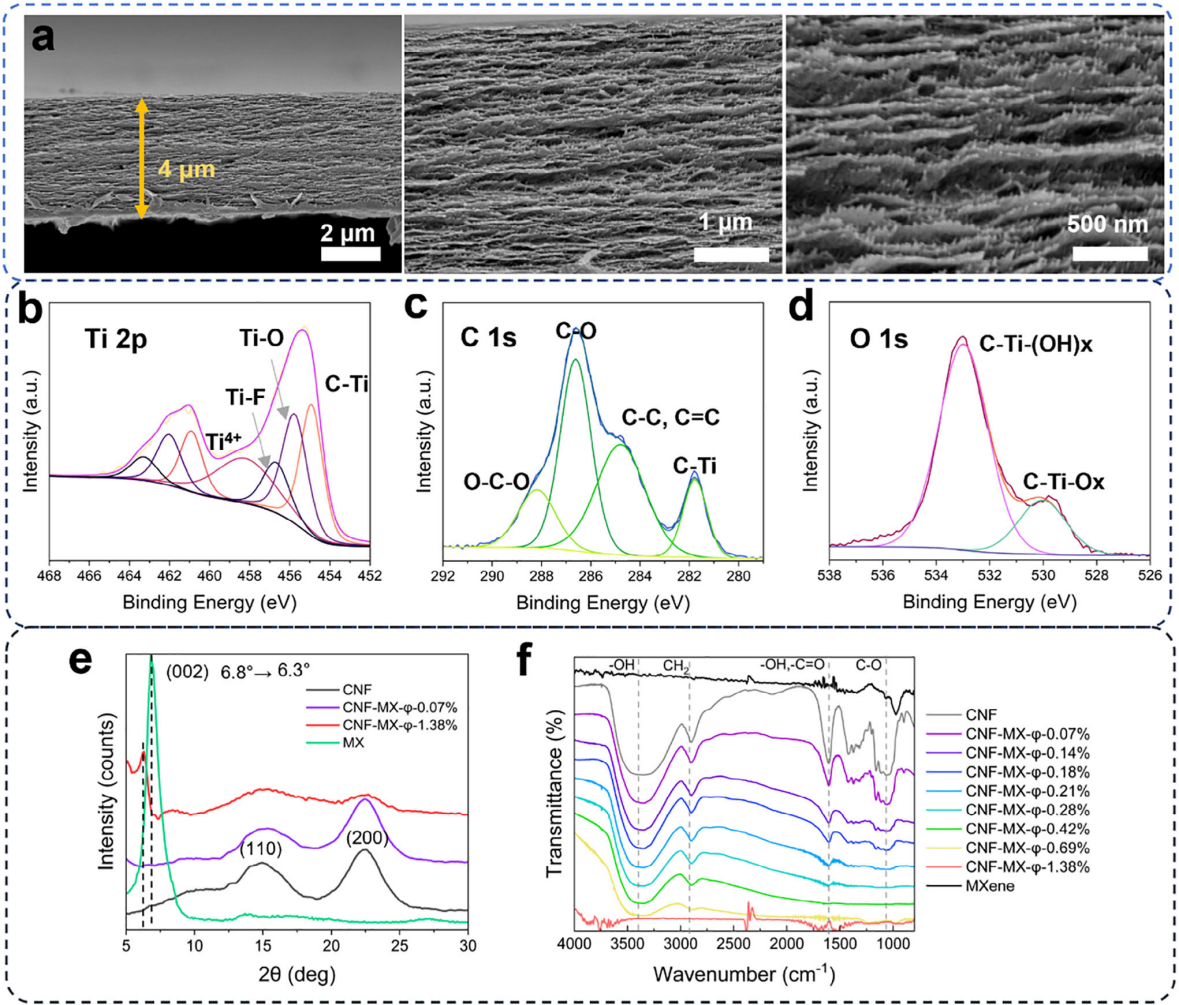
CNF‐MXene morphology, surface and compositional analysis. a) High‐resolution SEM cross‐sectional images of *CNF‐MX‐φ‐0.07%*. b–d) XPS narrow high‐resolution scans of the Ti 2p, C 1s and O1s of the *CNF‐MX‐φ‐1.38%*. e) XRD phase analysis for CNF, MXene, and their composite films *CNF‐MX‐φ‐0.07%* and *CNF‐MX‐φ‐1.38%*. f) FTIR spectra for CNF, MXene, and their composite films CNF‐MXene in all studied concentrations.

The fracturing of composite films shows well‐developed layered morphology, with nanofiber bundles of ≈50 nm diameter occupying 95+% of the volume (**Figure** **2**a).

The thickness of Ti_3_C_2_T*_x_* nanosheets and cellulose nanofibers dimensions were measured with AFM (Figure S4a,b, Supporting Information). MXene flake average thickness was 2 ± 1 nm, lateral size was within 2–8 µm, and CNFs of 1–2 µm long possess a diameter of 3 nm. MXene flakes were hard to observe in the SEM cross‐sections of CNF‐MX films due to flake dispersed distribution within a layered cellulose matrix without any signs of stacking and aggregations (Figure S2b, Supporting Information). We relate this phenomenon to the nanofibers assembling around the flakes stabilized by hydrogen bonding, as was demonstrated further for the different individual flakes .^[^ ^26^ ^]^ Dense bundle morphologies and reduced film porosity were observed for *CNF‐MX‐φ‐0.07%* and *CNF‐MX‐φ‐1.38%*, with increasing MXene content (Figure 2a; Figure S3, Supporting Information).

XPS survey spectra and high‐resolution scans were collected for CNF, MXene, and CNF‐MXene films (Figure 2b–d; Figure S5, Supporting Information). For CNF films, high‐resolution peak scans for C 1s are deconvoluted into components at 285.4 eV for C─C (carbon in a graphitic or aliphatic environment) and 287.1 eV for C─O (typical for ethers or alcohols in cellulose), while O 1s peak is positioned at 531.7 eV for C═O (carbonyl groups), and 533.5 eV for ─OH (hydroxyl groups), consistent with the oxygen functionality in cellulose (Figure S5b,c, Supporting Information).^[^ ^34^, ^35^ ^]^ In Ti_3_C_2_T*_x_* film C 1s spectra, C─C is located at 285 eV and a significant peak at 281.9 eV for C‐Ti, indicating the bonding between carbon and titanium in the MXene structure (Figure S5e, Supporting Information), while Ti 2p displays splitting of Ti 2p_3/2_ and Ti 2p_1/2_ states (Figure S5d, Supporting Information), and O 1s peaks were deconvoluted into C─Ti─O/(OH)_x_ at 533.5 eV (Figure S5f, Supporting Information).^[^ ^36^, ^37^ ^]^ The CNF‐MXene film shows C‐C, C‐O, and, most importantly, C‐Ti peaks at a binding energy of 281.8 eV (Figure 2b–d).

X‐ray data at various MXene loading shows a broad peak ≈22°, corresponding to the (200) plane of cellulose (Figure 2e).^[^ ^16^, ^38^ ^]^ MXene exhibits a characteristic sharp peak of (002) plane at 6.8° and shifts to 6.3° in *CNF‐MX‐φ‐1.38%*, reflecting an interlayer spacing increase from 12.9 to 14.16 Å. The peak shift indicates the bonding of the Ti_3_C_2_T*_x_* with cellulose nanofibrils rather than flake stacking and further confirms the absence of flake aggregation.^[^ ^21^ ^]^ Reduced intensity and sharpness are caused by very low MXene content. Furthermore, the (002) peak was not detectable for *CNF‐MX‐φ‐0.07%* film with the lowest MXene content.^[^ ^28^, ^30^ ^]^

FTIR analysis was conducted to monitor the bonding interactions within the CNF‐MXene films (Figure 2f). CNF films show broad hydroxyl stretching at around 3360 cm^−1^, bending at 1610 cm^−1^, and a C‐H stretching at 2900 and 1728 cm^−1^ C═O stretching, among others.^[^ ^39^ ^]^ The overlap of the Ti_3_C_2_T*_x_* and CNF peaks at some wavenumbers, particularly in regions where both materials have characteristic absorbances like O─H stretching, further confirms interactions between the MXene flakes and cellulose chains, altering the chemical environment of both components and indicating strong hydrogen bonding.^[^ ^21^, ^27^ ^]^

**Figure 3:**
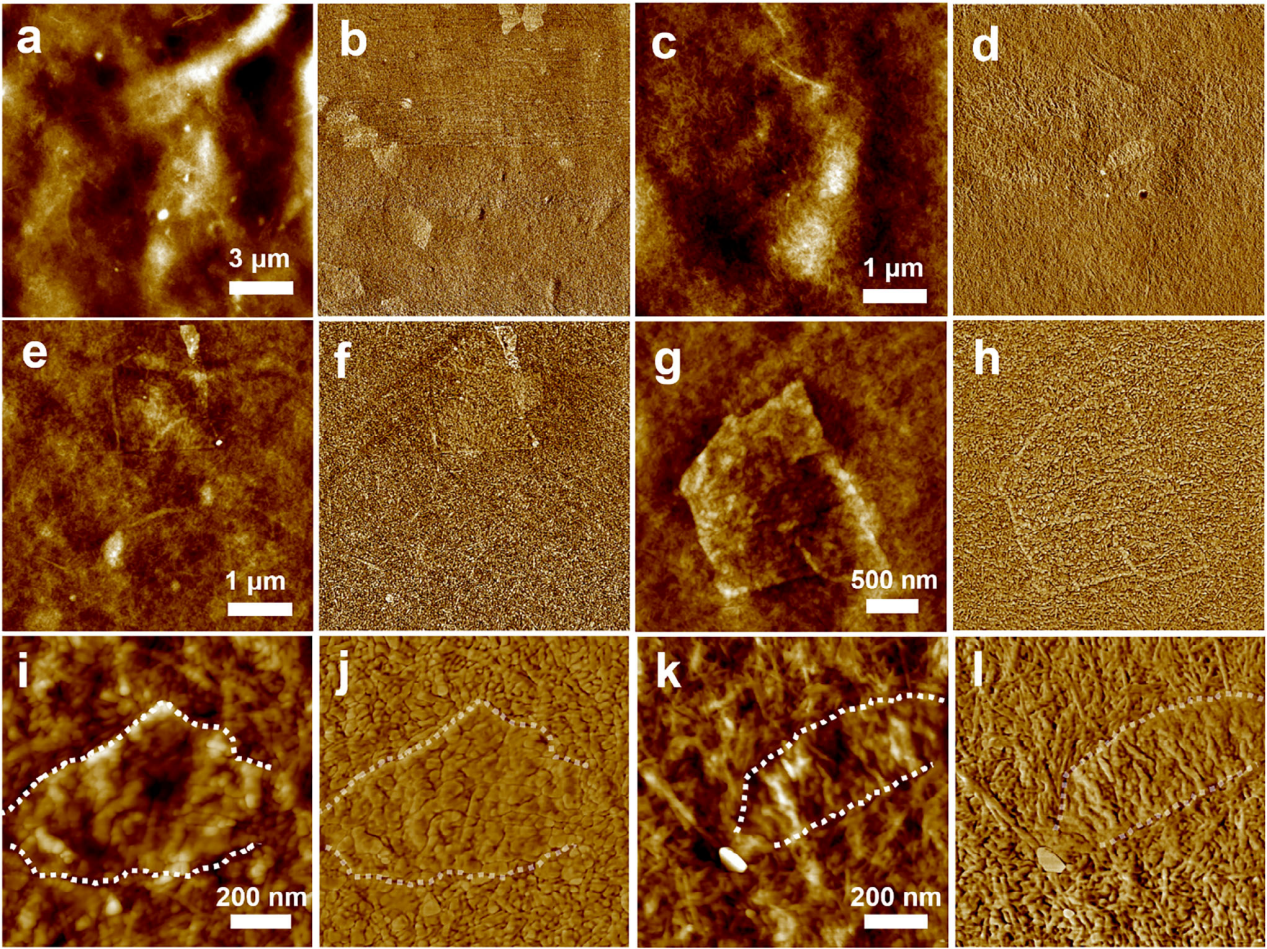
MXene flakes within the CNF surface of a CNF‐MXene film. a‐l, High‐resolution AFM topography (a,c,e,g,i,k) and phase (b,d,f,h,j,l) image pairs of various CNF‐MXene membrane surface areas, where (i‐j) show full top surface coverage, and k,l) partial top surface flake coverage outlined in white. Z‐scale: a) 100 nm, c,e,g) 50 nm, i,k) 20 nm; phase 20°.

The random bundled nanofiber morphology is observed via AFM for the CNF‐MXene film's surface across the whole surface area (**Figure** **3**). The surface roughness values of the composite films of Rq = 8.6 nm in a 5 × 5 µm area indicate a very smooth surface of films, as suggested earlier from optical reflectance measurements.

MXene flakes are distributed within CNFs and cannot be spotted easily on a surface (see Figure 3a,e). However, since MXene and CNF have different mechanical and adhesive properties, with MXene being stiffer, a distinct contrast between the two materials can be seen in phase images (Figure 3b,d). Flakes located beneath a thinner CNF layer can be detected with topography imaging and become hidden when situated deeper within CNF media, as seen in phase image (Figure 3e–h). High‐resolution AFM topography and phase images highlight surface contours and flake shapes embedded within CNF layers, revealing variations in coverage of the flakes under the top surface (Figure 3i–l), including full (Figure 3i–j) and partial coverage (Figure 3k,l) (also see Figures S6, S7, Supporting Information).

### 2.4 Optical Microscopy Observations

**Figure 4:**
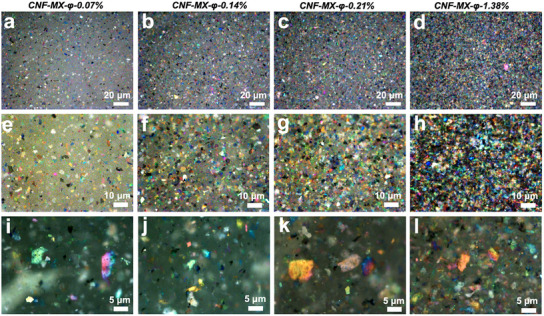
Structural colors of MXene flakes in CNF‐MXene films. Bright field optical microscopy of a,e,i) CNF‐MX‐φ‐0.07%, b,f,j) CNF‐MX‐φ‐0.14%, c,g,k) CNF‐MX‐φ‐0.21%, d,h,l) CNF‐MX‐φ‐1.38%.

Bright‐field optical microscopy images in reflection mode of CNF‐MXene films of 0.07%, 0.14%, 0.21%, and 1.36% MXene *φ*, captured at different magnifications, show a unique rich multicolor appearance with vivid red, orange, yellow, green, cyan, blue, magenta and purple colors of various individual flakes dispersed in the CNF matrix (**Figure** **4**).

A noticeable increase in the density and color intensity of the flakes can be observed with an increase in MXene loading from *φ‐0.07%* to *φ‐1.38%* (Figure 4). Additionally, *CNF‐MX‐φ‐0.07%* was recorded under several lighting scenarios with a digital microscope to observe surface features of the film via full and partial coaxial lightning, as well as transmittance, to highlight well‐dispersed flakes in the cellulose matrix (Figure S8, Supporting Information).

When observed directly through the optically transparent cellulose matrix, the MXene flakes appear as multicolored species with a range of vivid colors that are controlled by the subsurface depth location, thickness, composition, and orientation within the CNF matrix, as discussed below. Different thicknesses of the delaminated flakes are evident from transmission mode, signifying the existence of single to few‐layer stacks of MXene, where thicker flakes will produce brighter reflection colors (Figure S8a,d, Supporting Information). Larger flakes can show two colors that indicate a tilted flake within the CNF matrix (Figure 4i‐l). Optical images for CNF‐MXene film, MXene flakes, and CNF film deposited on a glass slide do not reveal the reflectance colors of individual flakes (Figure S9a–c, Supporting Information).

### 2.5 3D Optical Profile and Hyperspectral Imaging

**Figure 5:**
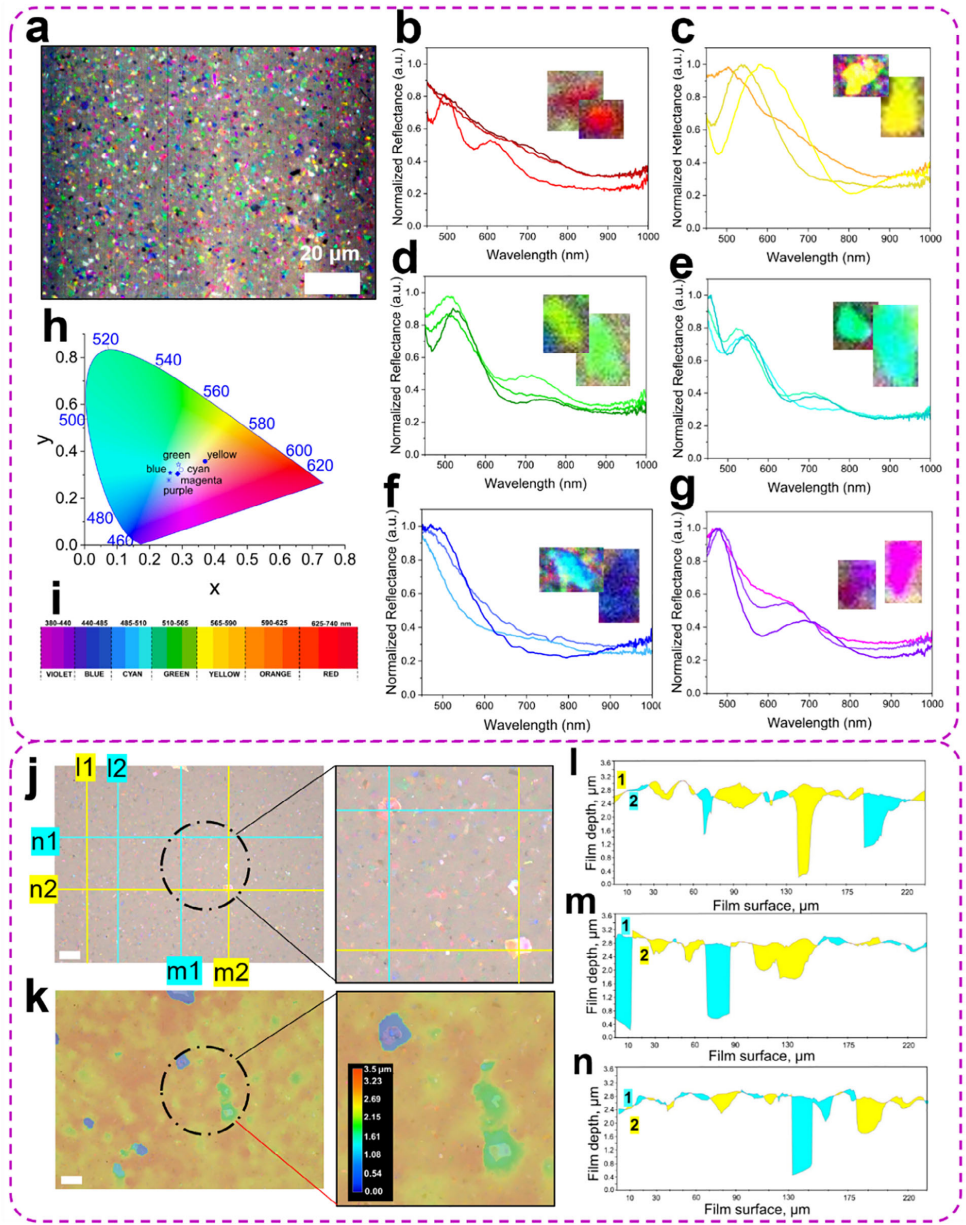
Structural colors of MXene flakes in CNF‐MXene hyperspectral and 3D profile analysis. a) Hyperspectral image of *CNF‐MX‐φ‐0.07%*. b–g) Normalized reflectance spectra obtained from hyperspectral imaging of each colored flake. Insets show areas with colored flakes where reflectance spectra were recorded. h,i) Colorimetric mapping of the obtained reflectance spectra with color locations in parametric space and spectral wavelength‐color relationship. j,k) Digital bright field optical microscopy image and respective 3D topography image with depth scale bar in black, obtained by stitching 300 images throughout the depth of *CNF‐MX‐φ‐0.07%* with blue and yellow line profiles cut through the surface z‐scale. l–n) Obtained line profiles across the film surface from (j,k).

Hyperspectral imaging was performed to investigate bright color reflections of individual flakes (**Figure** **5**a‐g). First, a regular optical bright field image of the selected area was collected, after which a hyperspectral image of the same area was collected (Figure 5a). The reflectance spectra were extracted from different regions and then normalized to the reflectance spectrum of the source (Figure 5b–g).

For further interpretation, the spectra are sorted by color with a representative spectrum. A peak in a spectrum of each flake in different colors represents wavelengths where the individual flake strongly reflects light. For example, on the spectra of the green flakes, three distinctive peak positions can be observed at 436 nm (violet), 528 nm (green), and a weaker broad 715 nm (red) peak with a slight interference contribution (Figure 5d). Main vivid peaks, however, fall into the blue‐green part of the spectrum, contributing to the overall bright green color appearance of the flake (Figure 5d). Similarly, cyan flakes fall into the blue‐green part of the spectrum, shifting toward a higher wavelength by 20 nm from green spectra with peaks at 454, 541, and 732 nm, resulting in cyan color (Figure 5e). Blue reflection spectra comprise one distinctive peak at 452 nm, shifting to 488 nm with increased flake size and brightness (Figure 5f). Red spectra have two peaks at 494 and 609 nm in blue and orange regions (Figure 5b), while magenta‐purple appearance shows stronger peaks at 476–581 nm in the blue region and weaker in 650–691 nm red region (Figure 5g).

The presence of two peaks that correspond to two different colors, blue and orange, instead of pure red peaks in the reflectance spectra of some flakes, can be related to the layered structure of the composite with different wavelengths reflected concurrently. Moreover, despite the fact that one type of MXene was used in this study, each flake, depending on the size, will still have a variation in the surface chemistry distribution. We speculate that modifying surface terminations of MXene, composition via synthesis methods, and/or functionalization might lead to various structural and color effects that can be investigated in the following studies. A broad peak in the NIR region of the green, cyan, and purple flakes furthermore suggests the structural and compositional complexity of the flakes’ surface caused by limited stacking, tilting, bending, folding, or cracking as especially visible for larger >4 µm flakes (Figure 4i–l).

To investigate the possible color‐depth relationship of individual MXene flakes within the nanocellulose matrix, 2D and 3D topography images were obtained with high‐resolution optical digital microscopy (Figure 5j,k). 3D‐colored image of the *CNF‐MX‐φ‐0.07%* film displays a depth of ≈3.6 µm, where the top surface is colored in red‐orange, and the bottom surface of the film at 0 µm is blue, as indicated on the scale bar (Figure 5k). Vertical (**l1, l2; m1, m2**) and horizontal (**n1, n2**) line profiles were plotted across the film to find the correlation of the subsurface position of individual flakes (Figure 5l–n). From the 3D profile, it is evident that colored flakes can be found at various depths. For instance, the dark green flakes are located within the top 100 nm. It is worth noting that bigger flakes are located deeper within the film as caused by the dynamic of VAF processing (Figure 5j,k). However, connecting each color of the flake to a specific subsurface depth position appeared difficult and required full‐color mapping and simulations.

### 2.6 FDTD Simulations and Color Mapping

**Figure 6:**
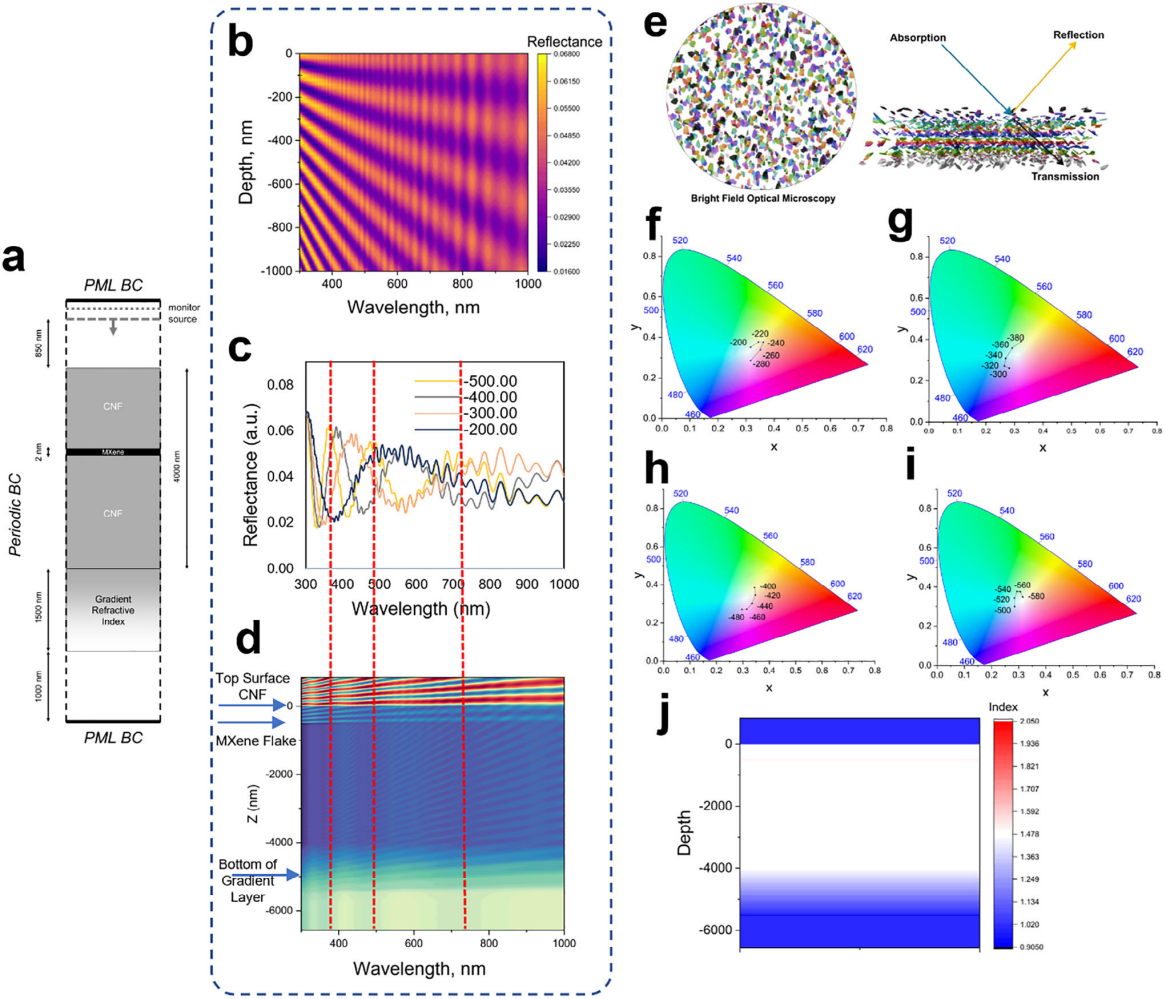
FDTD Simulations on color‐depth relationship for structural colors in CNF‐MXene. a) FDTD Simulation setup for Ti_3_C_2_T_x_ MXene flake placed within CNF layer of 4 µm thickness. b) A 2D plot of the simulated reflectance spectra. c) Respective reflectance obtained with varying depths of the MXene in CNF: −200, −300, −400, and −500 nm. d) FDTD model for the MXene flake embedded within a CNF layer at a depth of −500 nm with the periodicity of resonating waves evident in electric field distribution, which correlates with the reflectance spectra from the 500 nm depth shown in b. The reflectance peaks at 365, 473, and 718 nm correspond to 4, 3, and 2 resonating waves, respectively, between the top surface and the MXene flake. e) The schematic concept of the periodic color change observed in the depth of CNF‐MXene film. f–i) Colorimetric mapping of the reflectance spectra as obtained from FDTD simulations for varied flake depth range. j) The simulated refractive index profile of CNF‐MXene with MXene at −500 nm depth.

For FDTD simulations, a single MXene flake of 2 nm in thickness is positioned at varying subsurface depths within the 4 µm thick CNF matrix (**Figure** **6**a). The reflectance spectra were simulated for the depths of −200 to −500 nm from the top surface (indicated as a negative value from the zero level of the topmost surface). The 2D plot of the simulated reflectance provides a summary of the reflectance map across a depth range of 0 to −1000 nm from the top surface of the CNF‐MXene films (Figure 6b). This plot reveals that the number of photonic bandgaps appears within the CNF matrix, consequently changing the enhanced color of reflected light of individual flake “mirrors”, and signifying the appearance of periodic multicolor repeatedly throughout the depth positioning.^[^ ^40^ ^]^

First, we observed a single broad reflectance peak at ≈530 nm for the flake which is located at the depth of −200 nm (Figure 6c). As the depth increases, multiple reflectance peaks emerge in the blue and red regions of the spectrum: at 426 and 750 nm (−300 nm depth), at 385 and 563 nm (−400 nm depth), and at 364, 478, and 686 nm (−600 nm depth), showing systematic blue shift with increased depth (Figure 6c). These reflectance peaks occur with a high relative intensity and are overlapped by the air‐film interference effects as discussed below. The periodicity of the flake's color hues is evident through the colorimetric mapping of the simulated reflectance spectra at various flake depths (Figure 6f–i).

Notably, more vivid color hues are observed at depths ranging from −100 to −600 nm, with similar hues reappearing periodically at larger depths of flake location. Similar hues highlight the periodic occurrence of colors in the −280 to −260 nm region and the −460 to −440 nm region (Figure 6f,h). This phenomenon results from multiple peak appearances and shifts.

Since colorimetric coordinates are determined by color‐matching functions that integrate the reflectance spectra with the tristimulus spectra, even slight peak shifts can lead to dramatic color changes.^[^ ^41^ ^]^ For example, a color in the magenta‐red spectrum could correspond to depths of either −440 or −260 nm, making it difficult to pinpoint the exact flake location based solely on color. It is important to note that even a minor change in the flake's position, as small as 20 nm, can significantly affect the reflected color. Therefore, assigning an experimentally observed color to a very specific depth is challenging (Figure 5, also see Figure S10, Supporting Information).

At a greater depth in the CNF‐MXene composite, the reflected colors become less prominent due to numerous bandgaps beyond the −1000 nm producing multiple reflectance peaks superseding each other, which will place the colorimetric coordinates close to the white region of the CIE 1931 plot (Figure S11, Supporting Information). More drastic color shifts occur with greater depth differences. For instance, at a depth of −400 nm, two reflectance peaks appear in the visible range that correspond to the yellow region (Figure 6h). At −500 nm, the spectra display three photonic peaks resulting in colors within the cyan‐blue region (Figure 6i).

Next, the reflectance peaks observed in the CNF‐MXene can be explained by interference between the air‐film interface as well as at the CNF‐MXene interface. A specific case at a depth of −500 nm illustrates this phenomenon (Figure 6d). The electric field distribution across all wavelengths reveals variations in the periodicity of the wave across the spectrum. At wavelengths of 365, 473, and 718 nm, an integer number of wave periods is confined between the flake and the top air‐film interface, resulting in minimal field intensity at the upper interface. This configuration enhances reflection, as shown in Figure 6d and Figure S12a (Supporting Information). In contrast, at 418 nm, the wave does not have integer periods in the region between the flake and the top interface, which results in decreased reflectance (Figure S12b, Supporting Information).^[^ ^42^ ^]^ A gradient index layer at the bottom interface (Figure 6j) minimizes reflections from this boundary, thus having a negligible impact on the overall reflectance spectrum, resulting in low‐intensity thin‐film interference (Figure 6c).

This analysis underscores the importance of wave interference patterns and their relationship to the photonic structure of the CNF‐MXene film in determining its optical properties. The pattern discussed above for specific cases is consistent across all observed peaks and troughs in the optical reflectance. The periodicity of the color appearance is dependent on the flake's position and distance to the top surface within cellulose, producing a multispectral reflective multilayer thin film with the interplay of constructive and destructive interferences observed at a microscale for individual flakes (Figure 6e).

Overall, we suggest that MXene flakes act as metallic “mirrors” in the self‐assembled metal‐insulator‐metal layered structure, creating complex interactions of light with it, including absorption, transmission, reflection, and scattering within CNF dielectric layers, contributing to specific reflection colors of the individual flakes. The position and dimensions of the microcavities can be tailored by using various MXene flake lateral sizes, assembly conditions, and thicknesses of layered cellulose network, potentially allowing specific wavelengths of light to resonate within the cavity. Various thicknesses of MXene layers can increase the reflectivity of specific wavelengths, amplifying one specific color, while adjusting the periodicity of metal‐insulator‐metal structure can allow for precise control of the transmitted/reflected wavelength, allowing for advanced responsive optical materials development.

## 3 Conclusion

In conclusion, we fabricated flexible, robust, transparent free standing films with self‐assembled layered morphology and preferred orientation of MXene flakes parallel to the film surface in a cellulose nanofiber matrix. These CNF‐MX films demonstrate multispectral photonic structural colors of individual flakes ranging from violet to red combined with the overall high transparency of the films. We suggested that periodically distributed structural colors originate from constructive interference of the Ti_3_C_2_T*_x_* flakes confined at different depths and acting as reflective micromirrors within complex interference patterns at the air‐film interfaces. Self‐assembled metal‐dielectric stacks of MXene are arranged horizontally and dispersed within the CNF matrix, creating optical cavities that enhance or negate light interference at different wavelengths. Moreover, these thin films possess high flexibility and superior mechanical properties with elastic modulus near 10 GPa as will be elaborated in detail in a separate publication.

The discovery of this type of microscale structural coloration opens the path to designing responsive optical metamaterials based on MXenes and polymers by tailoring the optical microcavity's positions and dimensions, thus, creating new structural coloration functionalities with the potential for advanced multispectral photonic applications, photothermal phenomena, and optical communication. For future studies, we suggest that it is possible for these materials multispectral performance to be triggered by light irradiation for bio‐based applications like photothermal and photodynamic therapy and wound healing, as well as get response through external stimuli, such as electric or magnetic field, by modifying the micromirror function with different molecules and/or nanoparticles.

## 4 Experimental Section

### Cellulose Nanofibers (CNF)

A TEMPO‐mediated oxidation process was used to extract CNF from Bleached Kraft Pulp (International Paper).^[^ ^18^ ^]^ First, 1 g of softwood pulp was torn into small pieces, washed with Milli‐Q ultrapure deionized (DI) water, and placed into a beaker containing 100 mL DI water under magnetic stirring. Hereafter, 0.1 mmol TEMPO (2,2,6,6‐tetramethylpiperidine‐1‐oxyl) and 1 mmol sodium bromide (NaBr) were added to a wood pulp suspension, which was then oxidized using a 10 mmol sodium hypochlorite (NaClO, 12%) solution at room temperature while maintaining a pH 10 using 0.1 M NaOH stock solution at 500 rpm stirring rate for several hours. After oxidation, the mixture was washed with DI water and then sonicated for 30 min in an ice bath using a large tip sonicator, Qsonica Q125, with a 1/8′ diameter probe (40%amp, 10 min, 5on/5off) to separate the nanofibers. The obtained transparent mixture was centrifuged to remove any unexfoliated fibers at 10 000 rpm two times, resulting in a fine CNF dispersion of about 0.32–46 wt%.

### MXene Synthesis

The Ti_3_C_2_T*_x_* MXene was synthesized using a previously reported multi‐step process by selective wet‐chemical etching of Al from Ti_3_AlC_2_ MAX phase produced by Carbon Ukraine, Ltd, with particle size <40 µm.^[^ ^43^ ^]^ 1 g of HCl washed, dry Ti_3_AlC_2_ MAX phase was immersed in 20 mL of etchant and stirred at 300 rpm at 35 °C for 24 h. The etching solution contains a mixture of HF (48‐51 wt%, Acros Organics), HCl (37 wt%, Fisher Scientific), and DI water with a volumetric ratio of HF:HCl:H_2_O equal to 1:6:3. Multilayered Ti_3_C_2_T*_x_* MXenes were intercalated with LiCl (99%, Alfa Aesar) using 1 g of LiCl per 1 g of Ti_3_AlC_2_ MAX, dissolved in 50 mL of DI water, and stirred at 300 rpm at room temperatures for 24 h. The resulting solution was washed with DI water and centrifuged at 3500 rpm for 5 min. The supernatant was discarded, and the delaminated MXenes were redispersed by manual shaking. The washing cycles were repeated until the supernatants reached pH 6. Then, the colloidal solution was centrifuged at 3500 rpm for 60 min, and the supernatant containing single layer Ti_3_C_2_T*_x_* was collected. The resultant MXene was dispersed in water in chosen concentrations for further use and stored at −80 °C.

### CNF‐MXene Composite Film Fabrication

CNF‐MXene free‐standing thin films were fabricated by filtering the suspension of mixed CNF and MXene in water through a membrane via VAF. First, Ti_3_C_2_T*_x_* MXene in desired concentrations (0.005–0.1 mg mL^−1^) was added into CNF aqueous stock solution (0.32–0.46 wt%) and diluted to a total 10 mL volume with DI water, and then dispersed with handshaking in a glass vial. Afterward, the mixture was transferred using a 5 mL volume pipettor onto a Durapore 0.22 µm 47 mm PVDF membrane assembled in a filtration vacuum set‐up and allowed to filtrate water under vacuum pressure. The obtained films were then moved into a vacuum oven and kept at room temperature for 24 h to completely dry. The films can also be allowed to dry at ambient conditions overnight. Finally, the films are detached from the PVDF support. The samples were named according to the calculated *φ* volume fraction of MXene inside the CNF matrix, e.g., CNF‐MX‐φ‐x%, where MX‐φ‐x% signifies calculated volume fractions of MXene in composite, which were 0.07, 0.14, 0.21, 0.28, 0.42, 0.69, and 1.38% (See Tables S2, Supporting Information, for more details). Separately, MXene‐only and CNF‐only free‐standing films were prepared similarly from respective stock solutions.

### Characterization techniques

*Optical analysis methods*: A Shimadzu UV‐3600 Plus spectrophotometer was used to acquire UV–vis–NIR absorbance spectra and transmission spectra of MXene in water, and CNF‐MXene mixtures at various concentrations using quartz cuvettes with a 10‐mm light path and CNF‐MXene films were measured as‐synthesized using a solid sample holder in the range of 200–900 nm wavelength. Water or CNF spectra were recorded before data collection of the colloids, depending on the sample type. The absorbance data was recalculated to transmission using the formula T = 10^−A^, where T represents transmission and A – absorbance. Reflectance spectra of solid films were collected with an integrating sphere attachment, ISR‐603, with a 0° incidence angle in the range of 200–1000 nm.

Optical microscopy utilizes the Olympus BX51 to characterize the CNF‐MXene films' visual appearance and the MXene flakes' distribution within a transparent cellulose matrix in bright light unpolarized mode with light exposure of 50–200 ms. Bright‐field reflection hyperspectral imaging was performed using a CytoViva Hyperspectral Imaging System. The samples were illuminated using a 150W halogen light source, and the reflected light (low wavelength detection limit is 450 nm) was collected through a high numerical aperture objective lens. The system's spectrophotometer, integrated with a high‐resolution camera, captured the hyperspectral data cube, encompassing a range of wavelengths from 450 to 1000 nm. The samples were attached on a double‐sided scotch tape on a glass slide and placed on a motorized stage to enable precise scanning across the area of interest. Each pixel in the resulting hyperspectral image contains a full reflection spectrum, which was subsequently analyzed to extract detailed spectral information. Colorimetric mapping was based on the reflectance data obtained from hyperspectral imaging with the Origin Software (v2022). The Chromaticity Diagram plugin was used to estimate the CIE 1931 coordinates and generate the 3D color plots.

The Keyence VHX‐7000 digital microscope was used to construct optical 3D topography. Imaging was conducted under various lighting settings, including full coaxial, partial coaxial, transmission, and HDR enhancement. The microscope's 4K CMOS image sensor provided a large depth of field and high resolution, enabling detailed observation. Additionally, the 3D topography of the samples was constructed using the system's 100×100mm XYZ motorized stage.

Spectroscopic ellipsometry measurements were conducted to obtain the n and k refractive index values of MXene and CNF layers. For this purpose, the Woollam M‐2000U Ellipsometer was used. First, the silica glass substrate was fully characterized for transmittance, delta, and psi constants. After that, the MXene film on the same substrate was measured for delta, psi constants, and transmittance. Sequentially, the material was modeled as a linear superposition of two harmonic and one Drude oscillator, which provided the initial model n‐k values. The transmittance and ellipsometric experimental datasets were combined, and the model n‐k values were fitted for each wavelength until the best fit was reached.

*Surface and composition analysis methods*: Atomic force microscopy (AFM) was employed to examine the surface topography of the samples on a Bruker Dimension Icon microscope operated in tapping mode.^[^ ^44^ ^]^ Probes from Mikro‐masch, specifically the HQ:XSC11/Hard/Al BS model using cantilever C or D, depending on the sample, were used. The probe tips had nominal radii of <20 nm. All samples' scanning rates were 0.6 Hz, and the AFM images were captured at resolutions of 512×512 pixels. Image processing and analysis were conducted using Nanoscope Analysis or Gwyddion Software. Before scanning, as‐synthesized films were attached to double‐sided scotch tape on a glass slide, and separately, Ti_3_C_2_T_x_ MXene flakes in water, CNF, and CNF‐MXene mixture were drop cast on piranha cleaned Si wafers.

To determine the surface elemental composition, X‐ray photoelectron spectroscopy (XPS) was used with survey spectra and high‐resolution spectra using a Thermo Scientific Nexsa G2 X‐Ray photoelectron spectrometer, featuring an Al K‐alpha monochromated micro‐focused source with a 400‐micrometer spot size. Survey scan spectra were collected three times with binding energies ranging from 0 to 1350 eV in 1 eV increments, and the high‐resolution scans were collected ten times for each element in 0.1 eV steps. The obtained spectra were analyzed with Thermo Scientific Avantage Software.

ATR‐FTIR spectroscopy examined the chemical composition and molecular interactions using Bruker Vertex 70 system equipped with an A191/Q QuickLock baseplate with a solid sample holder. Spectra were collected in transmission mode with a resolution of 1 cm^−1^ and 100 scans per sample. Before sample deposition, 200 background scans were recorded.

*Morphology and phase analysis methods*: Scanning electron microscopy (SEM) was utilized to study the cross‐sections of the films, which were attached to a 90‐degree SEM stub to carbon tape and coated with a 10 nm layer of Au:Pd using a Q150V Plus Automatic Coater. The high‐resolution SEM images were collected using the 8230 FE‐SEM equipped with a cold field emission gun with secondary electron detection at 3 kV and a 5–15 mm working distance.

X‐ray diffraction (XRD) of the thin films was recorded on Rigaku Smartlab XE coupled with a HyPix‐3000 2D detector and X‐ray radiation of a Cu anode (wavelength 1.54 Å). X‐ray data were collected in parallel geometry beam mode using a 0 background sample holder at a 1deg/min scan rate in the 2θ range: 2–30° with a greasing angle of 0.3° and subsequently processed using SmartLab Studio II software.

### Simulations

Finite‐difference time‐domain (FDTD) simulations were conducted with the Ansys Lumerical v2023 R2.1 FDTD Software. The film was represented as a dielectric medium with a thin embedded MXene layer. A mesh override with 0.4 nm cell size was used in the flake region. Periodic boundary conditions were imposed on the directions perpendicular to the plane‐wave propagation vector, and a perfectly matched layer (PML) boundary condition was used for the simulation domain boundaries in the direction of the propagation.^[^ ^45^, ^46^ ^]^ A ‘Field and Power Monitor’ was placed to measure the reflected signal. The parametric sweep was done with a 20 nm depth step for the MXene layer while measuring the reflectance spectra.

The MXene real and imaginary refractive indices were fitted for FDTD simulation via the built‐in parametric model fit by using the refractive index *n*_MXene_ and extinction coefficient *k*_MXene_ data obtained from ellipsometry, and the CNF was represented as a dielectric with a constant real refractive index *n*_CNF_ = 1.47 and imaginary part *k*_CNF_ = 0. The total film composite thickness is *d*_film_ = 4 µm, the MXene layer is *d*_MXene_ = 2 nm, and a bottom layer with gradient refractive index is *d*_grad_ = 1.5 µm. The layer with gradient refractive index *n*_grad_ from 1.47 to 1 was placed immediately after the film to reproduce the bottom surface roughness and anti‐reflection. Parametric sweeps with varying depths were performed to explore the effect of MXene flake depth on the film's optical properties – reflectance spectra and perceived colors. Colorimetric mapping was used to interpret the obtained FDTD reflectance spectra.

## Conflict of Interest

The authors declare no conflict of interest.

## Supporting information



Supporting Information

## Data Availability

The data that support the findings of this study are available in the supplementary material of this article.
