# Supplementary material for: Multispectral Photonic Structural Colors via Enhanced Interfacial Interference of Ultrathin Cellulose Nanofiber/MXene Films
Source: Adv Sci (Weinh). 2025 Apr 7;12(23):2500953. doi: 10.1002/advs.202500953 (PMC12199417; doi:10.1002/advs.202500953)
Supplement: Supplementary file 1 — Supporting Information [file ADVS-12-2500953-s001.pdf]

ADVANCED SCIENCE
Open Access

Supporting Information

for *Adv. Sci.*, DOI 10.1002/advs.202500953

Multispectral Photonic Structural Colors via Enhanced Interfacial Interference of Ultrathin Cellulose Nanofiber/MXene Films

*Valeriia Poliukhova, Botyo Dimitrov, Justin Brackenridge, Laura Mae Killingsworth, Iryna Roslyk, James Fitzpatrick, Yury Gogotsi and Vladimir V. Tsukruk**

Supporting Information

Multispectral Photonic Structural Colors via Enhanced Interfacial Interference of Ultrathin Cellulose Nanofiber/MXene Films

Valeriia Poliukhova[1], Botyo Dimitrov[1], Justin Brackenridge[1], Laura Mae Killingsworth[1], Iryna Roslyk[2], James Fitzpatrick[2], Yury Gogotsi[2], Vladimir V. Tsukruk[1,*]

[1] School of Materials Science and Engineering, Georgia Institute of Technology, Atlanta, Georgia 30332, United States

[2] A. J. Drexel Nanomaterials Institute and Department of Materials Science and Engineering, Drexel University, Philadelphia, Pennsylvania 19104, United States

CNF-MXene membrane films fabricated via vacuum-assisted filtration (VAF) were named according to the MXene volume fraction φ in the composited material, from 0.07 to 1.38%, respectively. With the increase in the MXene loading, the transmittance values across different wavelengths gradually decrease from 85% to 5% at 850 nm, 82% to 6% at 650 nm, 77% to 3.7% at 450 nm, and 63.7% to 0.2%, at 350 nm. (**Table S1**). The calculation of MXene φ and wt% in CNF-MXene films is presented in **Table S2**. Before film fabrication, CNF-MXene suspensions in studied concentrations were recorded with a UV-Vis spectrophotometer, and the obtained transmittance values of solutions can be observed in **Table S3**, which, on average, were 10% lower than the transmittance values recorded of solid films (**Table S1**). MXene concentration was determined based on UV-Vis measurements and the Beer-Lambert law with absorbance values from 754 nm of diluted MXene stock concentration from 0.005 mg/mL to 0.03 mg/mL. The absorbance and transmittance of various MXene concentrations in water are shown in **Figure S1**.

The cross-sections of layered cellulose bundles assembled in a layer-by-layer fashion are shown in **Figure S2a**. In CNF film, the layered fiber morphology of cellulose randomly bundled together is distinctive, while MXene film shows a typical lamellar structure (**Figure S2a-b**). Notably, MXene flakes are not detectable in a cross-sectional view of CNF-MX-φ-1.38% film with the highest content of MXene due to bonding with CNF and full coverage of the flake's

surface (**Figure S3**). The MXene flakes and cellulose fiber dimensions were measured as synthesized via high-resolution atomic force microscopy (AFM) drop-casted onto a Si-wafer, shown in **Figure S4**. $Ti_3C_2T_x$ thickness of 2±1 nm was measured via profile cut in 3 different regions of the flake topography, and lateral size was within 2 - 8 μm (**Figure S4b**). Cellulose nanofibers had a diameter of 3 nm and 1-2 μm length (**Figure S4d**).

XPS survey spectra are shown for CNF, $Ti_3C_2T_x$ MXene, and CNF-MXene films (**Figure S5a**) with high-resolution scans for individual components CNF (**Figure S5b-c**) and MXene (**Figure S5d-f**). CNF-MXene film surfaces are shown with AFM topography with various flake positions within CNF layers (**Figure S6**). Only the edges of the flakes embedded into CNF layers can be observed in some areas (**Figure S6a,b,g,h**) and are not visible at all when on top of the CNF layer's surface in topography, but are evident in phase (**Figure S6c,d**). When the flake is within the top surface of CNF, it can be observed from both topography and phase images (**Figure S6e,f)**. Interestingly, when the CNF-MXene mixture prior to film fabrication was drop-casted onto Si wafers, most of the flakes fell on top of the CNF surface rather than being embedded within CNF matrix (**Figure S7**).

CNF-MX-φ-0.07% surface is shown under bright field (BF) optical microscopy in different lightning settings to highlight the smoothness of the surface (**Figure S8**). The homogeneous distribution of the flakes can be observed, while some of the flakes appear brighter than others on the BF image (**Figure S8a**). This is due to the different thicknesses of delaminated flakes, where thicker stacks appear darker in optical transmission settings, and thinner flakes are deemed to have a transparent green hue (**Figure S8d**). The smooth film surface is apparent from partial coaxial lightning settings and HDR enhancement features of the optical microscope, also showing random fiber morphologies bundled on the top surface (**Figure S8b,c**).

The uniqueness of structural colors of MXene flakes in cellulose media discovered in this work has been proved by measuring individual components and shown in optical microscopy images in **Figure S9**. MXene film has a typical wrinkled surface in purple opaque color (**Figure S9a**), while cellulose nanofibers drop-casted onto a glass slide randomly bundle together with no distinct broad color regions (**Figure S9b**), and MXene flakes in water drop-casted onto a glass slide exhibit only white reflection color (**Figure S9c**).

Additional control experiments were conducted to measure the different dielectric layer heights that produce diverse reflection colors. Each layer was dried separately on a glass substrate before depositing the next layer. A layer of MXene flakes deposited on top of the CNF layer exhibited no difference in color, only white reflection (**Figure S10a**). However, with an additional CNF layer dried on top of the MXene layer, a gradient color appears due to the nature of water evaporation from cellulose suspension, forming the coffee-ring effect and signifying different dielectric layer thickness that alters the reflection color of the MXene flakes (**Figure S10b,c-f**). AFM measurements were conducted on the colored stacks of CNF-MXene-CNF with the brightest reflection color regions and showed subtle differences in color and thicknesses of the combined layer (**Figure S10g-k**). The measured thicknesses from the profile cut on AFM topography were 25 nm for magenta, 30 nm for purple, 35 nm for cyan, and 37 nm for green reflected CNF-MXene-CNF stacks, suggesting multiple MXene flakes in between CNF layers altering the displayed reflection color. These results also suggest that various reflection colors of the individual flakes in CNF-MXene films repeat periodically within the total thickness of a 4 μm composite film.

FDTD simulations with a single MXene flake placed within the dielectric CNF matrix are performed by varying the flake depth, as discussed in the main text. At greater depths than 500 nm, the colors become less prominent, and at depths approaching -1000 nm, they are close to white (**Figure S11**). Beyond -1000 nm, the bandgaps in the visible range are numerous (**Figure S11a-c**), causing a white color appearance due to multiple reflectance peaks, which will place the colorimetric coordinates close to the white region of the CIE 1931 plot (**Figure S11d-f**).

The reflectance peaks shown with the MXene flake placed at -500 nm depth from top surface (main text **Figure 6d**) can be attributed to the wave interference occurring between the top CNF-air interface and the CNF-MXene interface. The E-field plot for all wavelengths showed variation in the periodicity of the wave across the spectrum. For the peaks at 365, 473, and 718 nm, there was an integer number of confined wave periods between the flake and the top air-CNF interface. This resulted in a very low E-field intensity at the top interface of the film, which in turn produced an enhanced reflection at the peak reflectance wavelength of 365 nm (**Figure S12a**). Conversely, the trough wavelength of 418 nm, for example, did not show wave periodicity in the region between the flake and the top interface, which yields a decreased reflectance (**Figure S12b**).

Supplementary Figures

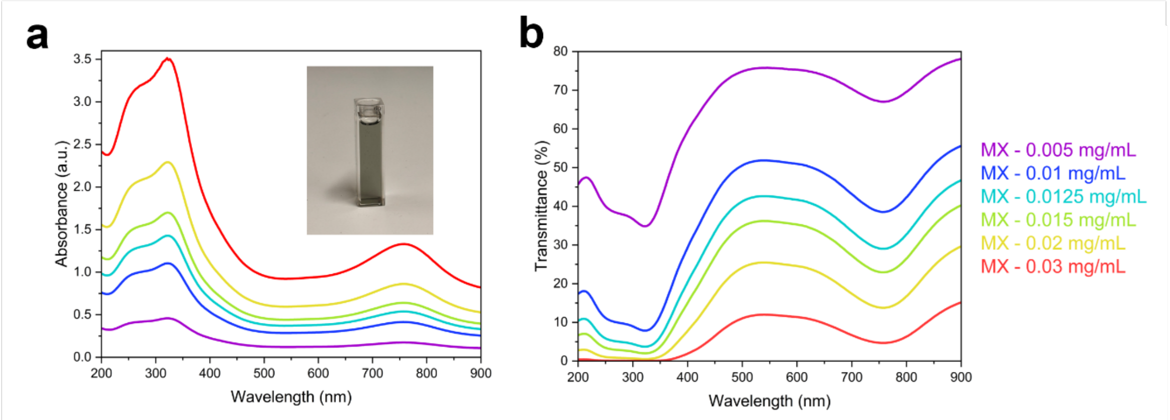

Figure S1. UV-Vis-NIR absorbance (a) and transmittance spectra (b) of MXene dispersed in water in various concentrations, with the inset showing quartz cuvette used for the measurement.

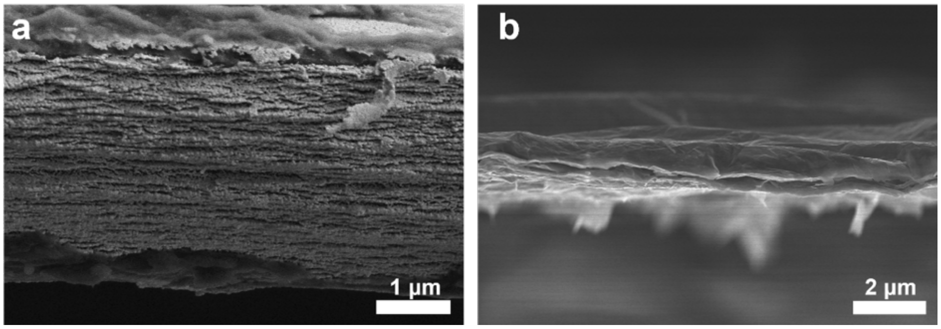

Figure S2. High-resolution SEM cross-sectional images of the CNF-only film (a) and MXene-only film (b) fabricated via vacuum-assisted filtration.

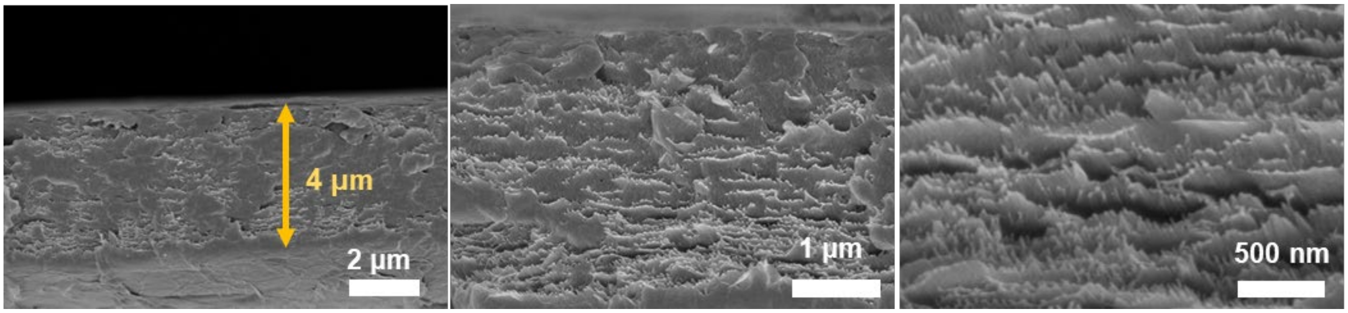

Figure S3. High-resolution SEM cross-sectional images of *CNF-MX-φ-1.38%* showing layered morphology.

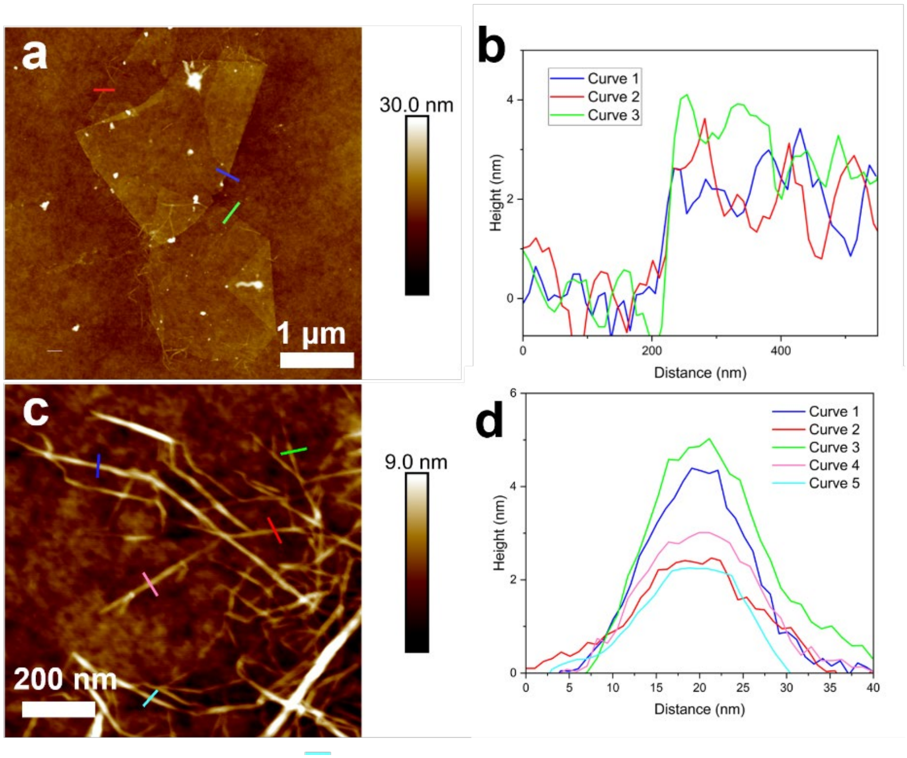

Figure S4. AFM topography images and the corresponding height profiles of the as-synthesized $Ti_3C_2T_x$ MXene (a, b) and CNF drop-casted on Si wafer (c, d), respectively.

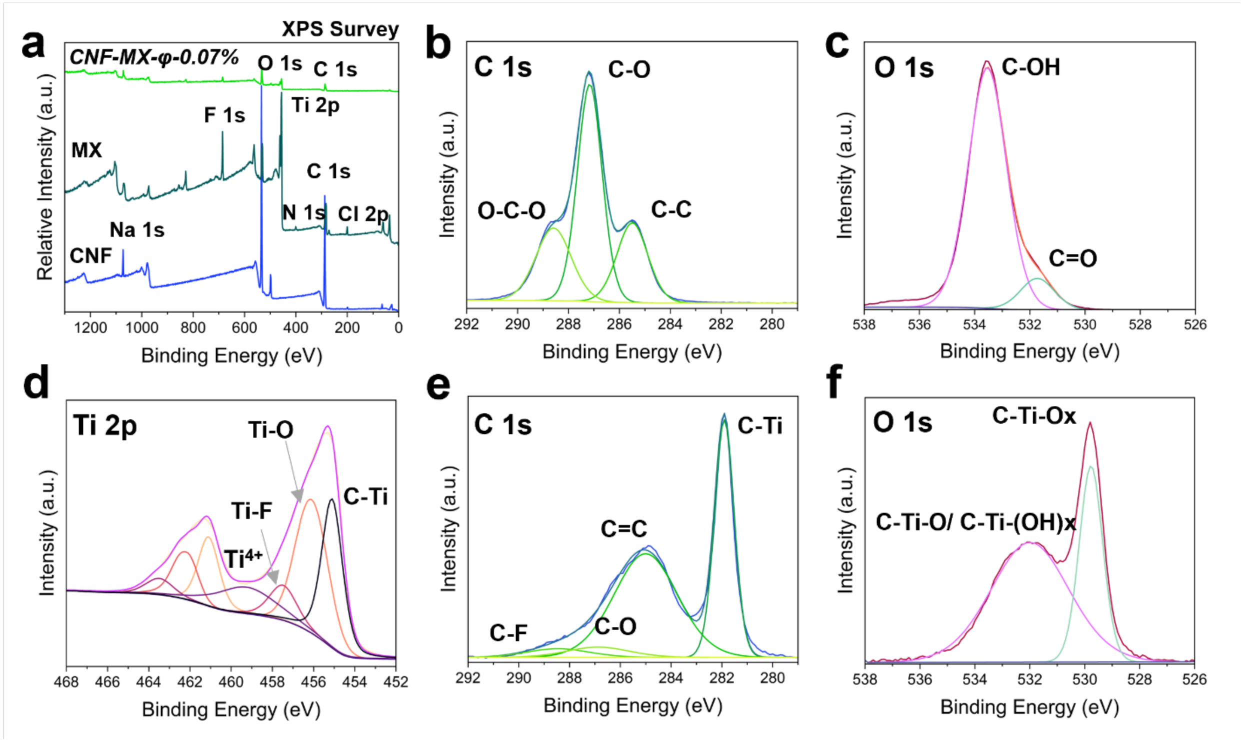

Figure S5. XPS survey spectra of (a) CNF, $Ti_3C_2T_x$ MXene, and *CNF-MXene* free-standing films, with XPS narrow high-resolution scans of the C 1s and O1s of the CNF film (b,c); Ti 2p, C 1s and O1s of the $Ti_3C_2T_x$ MXene film (d,e,f).

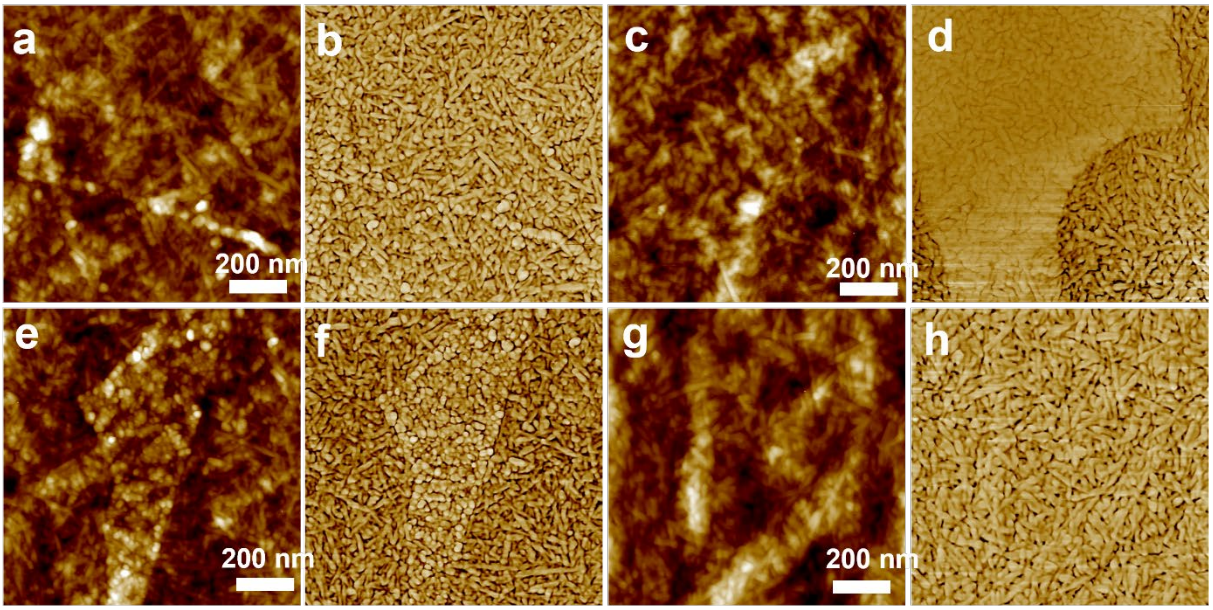

Figure S6. AFM topography and phase image pairs (a,b; c,d; e,f; g,h) of CNF-MXene film surfaces showing various flake positions within CNF layers and nanofiber morphology; Z-scale: 20 nm; phase 40°.

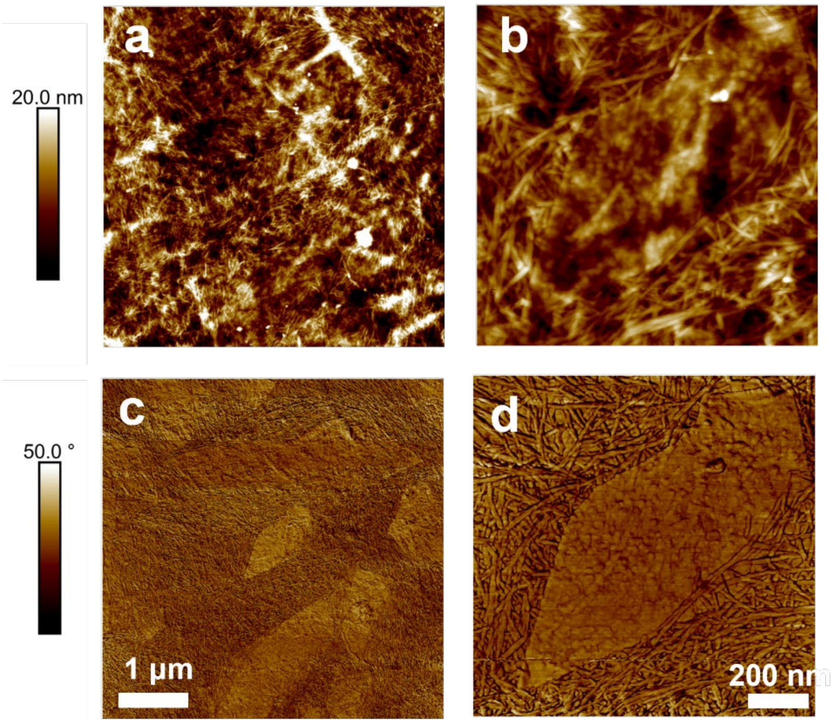

Figure S7. AFM topography and phase images (a,c; b,d) of CNF-MXene mixture drop-casted onto Si wafers, scale bar 1 μm (a,c) and 200 nm (b,d); Z-scale 20 nm, phase 50°.

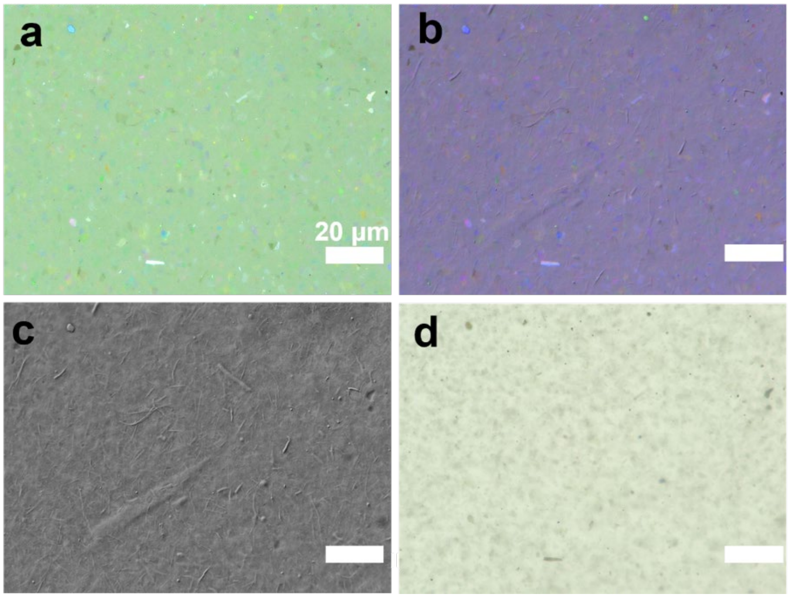

Figure S8. Bright-field optical microscopy of the *CNF-MX-φ-0.07%* surface in a different lightning setting (a) full coaxial light, (b) partial coaxial, (c) HDR enhancement, (d) transmission light, scalebar 20 µm.

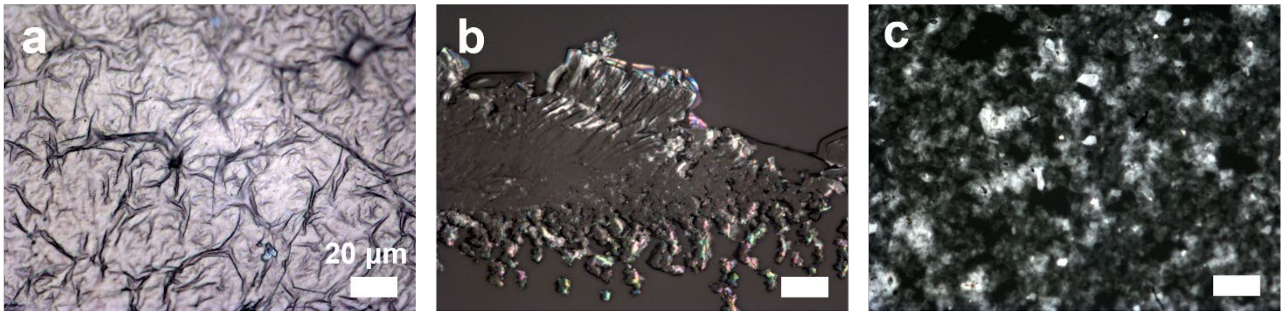

Figure S9. Bright-field optical microscopy of the (a) MXene film fabricated via VAF, (b) cellulose nanofibers drop-casted onto a glass slide, and MXene flakes in water drop-casted onto a glass slide at 50x magnifications, scalebar 20 µm.

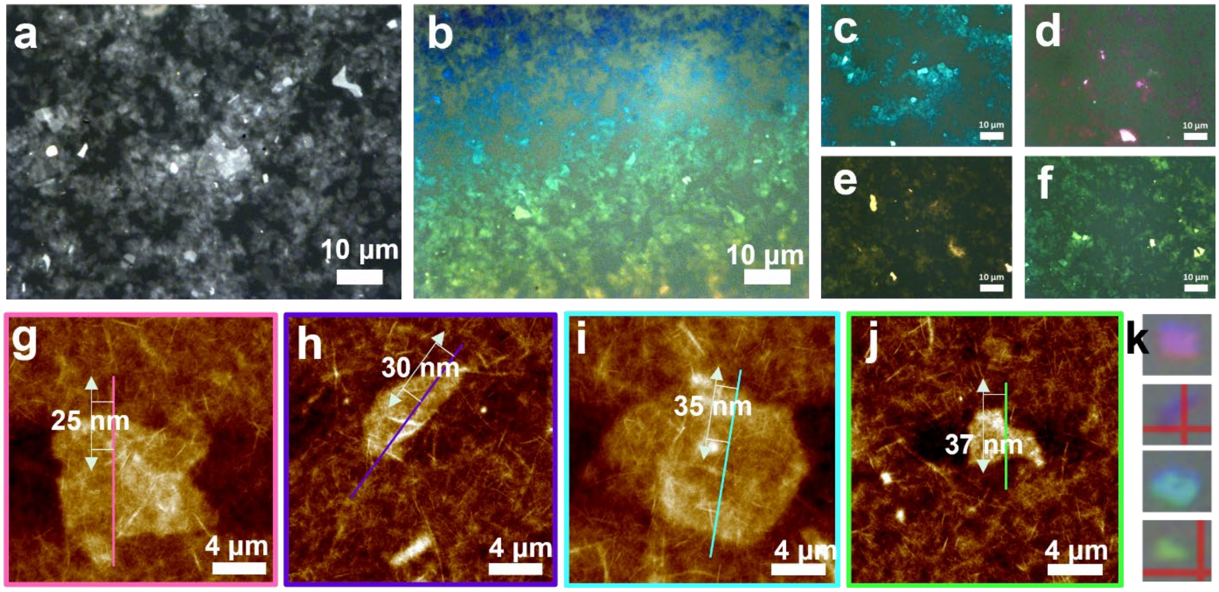

Figure S10. Bright-field optical microscopy of CNF/MXene (a) CNF/MXene/CNF (b-f) layers deposited onto the glass surface showing different color reflections depending on the top CNF thickness, and AFM images (g-j) measured on the region of interest of specific reflection colors (k) visible on the glass substrate to measure the thickness of the CNF/MXene/CNF stacks.

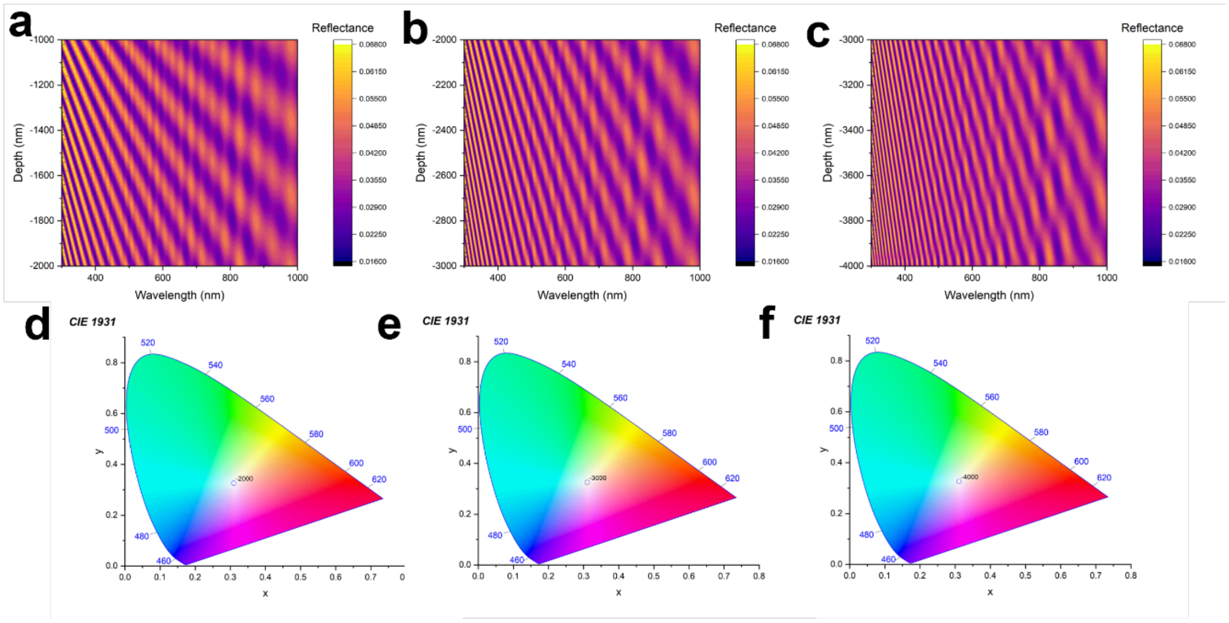

Figure S11. FDTD Simulations. 2D contour plots of the simulated reflectance for MXene varied depth placement within the CNF layer in CNF/MXene composite: from -1000 to -2000 nm (a), -2000 to -3000 nm (b), and -3000 to -4000 nm (c) and CIE 1931 colorimetric plots for -2000 nm (d), -3000 nm (e), and -4000 nm (f) showing the depletion of color when the depth of the MXene flake exceeds 1000 nm within CNF layer.

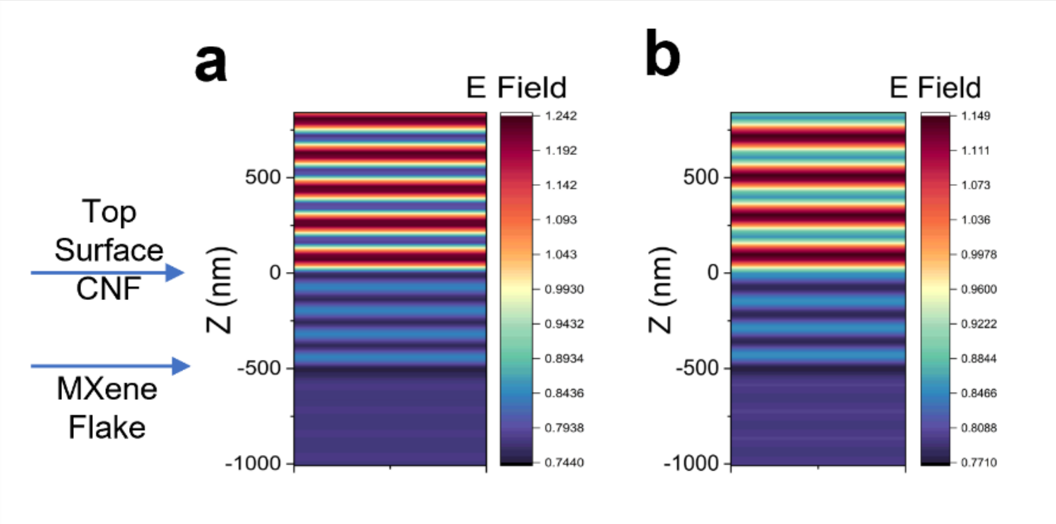

Figure S12. FDTD model for a $Ti_3C_2T_x$ MXene flake embedded within a CNF layer at a depth of -500 nm with electric field distribution on panels (a) and (b) showing the electric field at the peak reflectance wavelength of 365 nm and at the trough wavelength of 418 nm, respectively.

Supplementary Tables

Table S1. CNF-MXene thin films transmittance values in UV, visible, and Near-IR regions at different MX volume fractions *φ*, noted as *CNF-MX-φ-x*.

Wavelength, nm	CNF	φ-0.07%	φ-0.14%	φ-0.18%	φ-0.21%	φ-0.28%	φ-0.42%	φ-0.69%	φ-1.38%
350	87.8%	63.4%	36.5%	28.9%	21.4%	12.8%	4.7%	1.5%	0.2%
450	89%	77%	60.4%	54.3%	43.5%	33%	25.9%	16.6%	3.7%
650	90.6%	82.1%	66%	60.8%	53.1%	43.2%	31.9%	20%	6%
850	90%	85%	65.0%	60.6%	55.2%	43.8%	30.5%	19.1%	5.2%

Table S2. CNF-MXene composite films volume fraction with *φ* and wt% calculation.

Sample	MX C, mg/mL	MX mass in 1 film, mg	MX Volume, cm^3	Mx φ (Volume fraction), %	CNF, φ, %	MX wt%	CNF wt%
CNF-MX-φ-0.07%	0.005	0.02	0.000005	**0.070**	99.93	0.2	99.8
CNF-MX-φ-0.14%	0.01	0.04	0.000010	**0.140**	99.86	0.4	99.6
CNF-MX-φ-0.18%	0.0125	0.05	0.000012	**0.175**	99.82	0.5	99.5
CNF-MX-φ-0.21%	0.015	0.06	0.000014	**0.210**	99.79	0.6	99.4
CNF-MX-φ-0.28%	0.02	0.08	0.000019	**0.280**	99.72	0.8	99.2
CNF-MX-φ-0.42%	0.03	0.12	0.000029	**0.420**	99.58	1.19	98.81
CNF-MX-φ-0.69%	0.05	0.2	0.000048	**0.698**	99.30	1.96	98.04
CNF-MX-φ-1.38%	0.1	0.4	0.000095	**1.387**	98.61	3.85	96.15

Table S3. CNF-MXene solutions transmittance values in UV, visible, and Near-IR regions were recorded in quartz cuvette before the film fabrication.

Wavelength, nm	CNF	CNF_MX-φ-0.07%	φ-0.14%	-φ-0.18%	φ-0.21%	φ-0.28%	φ-0.42%	φ-0.69%	φ-1.38%
350	95.1%	44.7%	10.9%	6.15%	3.08%	1.22%	0.12%	0.003%	0.001%
450	98.2%	71.6%	40.5%	32.2%	24.3%	16.6%	6.73%	1.08%	0.022%
650	99.6%	76.5%	47.6%	38.8%	31%	22.5%	10.2%	2.15%	0.07%
850	99.9%	77.5%	49.61%	41.8%	32.7%	24.4%	12.5%	2.81%	0.11%
